# Decoding the Interdependence of Multiparametric Magnetic Resonance Imaging to Reveal Patient Subgroups Correlated with Survivals^1^^2^

**DOI:** 10.1016/j.neo.2019.03.005

**Published:** 2019-03-31

**Authors:** Chao Li, Shuo Wang, Pan Liu, Turid Torheim, Natalie R. Boonzaier, Bart RJ van Dijken, Carola-Bibiane Schönlieb, Florian Markowetz, Stephen J. Price

**Affiliations:** * Cambridge Brain Tumor Imaging Laboratory, Division of Neurosurgery, Department of Clinical Neurosciences, University of Cambridge, Addenbrooke's Hospital, Cambridge, UK; † Department of Neurosurgery, Shanghai General Hospital (originally named Shanghai First People's Hospital), Shanghai Jiao Tong University School of Medicine, China; ‡ Department of Radiology, University of Cambridge, Cambridge, UK; § The Centre for Mathematical Imaging in Healthcare, Department of Pure Mathematics and Mathematical Statistics, University of Cambridge, UK; ¶ Cancer Research UK Cambridge Institute, University of Cambridge, Cambridge, UK; # CRUK & EPSRC Cancer Imaging Centre in Cambridge and Manchester, Cambridge, UK; ** Developmental Imaging and Biophysics Section, Great Ormond Street Institute of Child Health, University College London, London, UK; †† Department of Radiology, University Medical Center Groningen, University of Groningen, Groningen, The Netherlands; ‡‡ Wolfson Brain Imaging Centre, Department of Clinical Neurosciences, University of Cambridge, Cambridge, UK

## Abstract

Glioblastoma is highly heterogeneous in microstructure and vasculature, creating various tumor microenvironments among patients, which may lead to different phenotypes. The purpose was to interrogate the interdependence of microstructure and vasculature using perfusion and diffusion imaging and to investigate the utility of this approach in tumor invasiveness assessment. A total of 115 primary glioblastoma patients were prospectively recruited for preoperative magnetic resonance imaging (MRI) and surgery. Apparent diffusion coefficient (ADC) was calculated from diffusion imaging, and relative cerebral blood volume (rCBV) was calculated from perfusion imaging. The empirical copula transform was applied to ADC and rCBV voxels in the contrast-enhancing tumor region to obtain their joint distribution, which was discretized to extract second-order features for an unsupervised hierarchical clustering. The lactate levels of patient subgroups, measured by MR spectroscopy, were compared. Survivals were analyzed using Kaplan-Meier and multivariate Cox regression analyses. The results showed that three patient subgroups were identified by the unsupervised clustering. These subtypes showed no significant differences in clinical characteristics but were significantly different in lactate level and patient survivals. Specifically, the subtype demonstrating high interdependence of ADC and rCBV displayed a higher lactate level than the other two subtypes (*P* = .016 and *P* = .044, respectively). Both subtypes of low and high interdependence showed worse progression-free survival than the intermediate (*P* = .046 and *P* = .009 respectively). Our results suggest that the interdependence between perfusion and diffusion imaging may be useful in stratifying patients and evaluating tumor invasiveness, providing overall measure of tumor microenvironment using multiparametric MRI.

## Introduction

Glioblastoma represents the most common brain malignancy, characterized by treatment resistance and poor outcome [1]. The remarkable interpatient variation of glioblastoma poses significant challenges to treatment stratification [2].

Tumor angiogenesis results in aberrant microvasculature in glioblastoma, which is typically inefficient in resource delivery and may induce heterogeneous blood flow [3]. In the meanwhile, cellularity significantly varies within the tumor, and high or low cellularity can exist in either sufficiently or poorly perfused subregions [4]. The spatial variations of tumor vascularity and cellularity can reflect the heterogeneous tumor microenvironment, which may be associated with patient treatment response [5]. For clinical decision making of individual patients, a systematic method to evaluate the overall tumor microenvironment is crucial.

Multiparametric magnetic resonance imaging (MRI) describes complementary properties of tumor physiology. Particularly, the relative cerebral blood volume (rCBV) calculated from perfusion imaging can measure the tumor vascularity and is correlated to the cellular proliferation [6]. The apparent diffusion coefficient (ADC) calculated from diffusion imaging can describe the tissue microstructure by measuring the microscopic water mobility [7]. Therefore, an integrated analysis of rCBV and ADC shows potential in evaluating tumor microenvironment by incorporating the information regarding tumor microstructure and vasculature [4].

Tumor habitat imaging is an emerging method of integrating multiparametric MRI, which uses thresholding intensity of perfusion and diffusion imaging to identify the local overlapping habitats [8], [9]. These habitats, however, are established to reveal the intratumoral various subregions and may be insufficient to provide the global information for individual patient evaluation. Instead, the overall evaluation of tumor microenvironment may potentially be enabled by investigating the interdependence between rCBV and ADC, describing vascularity and cellularity, respectively. However, the parametric model fitting of this interdependence is significantly challenged by the distinct marginal distributions of perfusion and diffusion imaging.

The copula transform is a statistical method to describe the interdependence of random variables by modeling the multivariate probability distribution [10] (Supplementary material 1 demonstrates the theoretical details of the method). In this study, we leveraged the copula transform to obtain the joint distribution of ADC and rCBV, from which discretized second-order features were extracted to characterize the interdependence between ADC and rCBV.

The purpose of this study was to investigate the utility of the interdependence between ADC and rCBV for evaluating tumor microenvironment and stratifying patients. Our hypothesis is that the interdependence among advanced imaging modalities may reflect tumor microenvironment and offer prognostic value for glioblastoma patients.

## Materials and Methods

### Patients

This study was approved by the local institutional review board. Informed written consent was obtained from all patients. Patients with a radiological diagnosis of *de novo* supratentorial glioblastoma were prospectively and preoperatively recruited for maximal safe surgical resection from July 2010 to August 2015. Exclusion criteria include the history of previous cranial surgery or radiotherapy/chemotherapy or contraindication for MRI scanning. All patients were required to have a good performance status (World Health Organization performance status 0-1). Preoperative MRI and postoperative histology were performed on all patients. All imaging and histological data were collected prospectively. A flowchart demonstrating patient recruitment is in Supplementary material 2.

### MRI Acquisition

All MRI sequences were performed at a 3-T MRI system (Magnetron Trio; Siemens Healthcare, Erlangen, Germany) with a standard 12-channel receive-head coil. MRI sequences included: postcontrast T1-weighted, T2-weighted, diffusion tensor imaging (DTI) with an inline ADC calculation using *b* values of 0-1000 s/mm^2^, dynamic susceptibility contrast-enhancement (DSC), and multivoxel two-dimensional ^1^H-MRS chemical shift imaging (CSI). Scanning parameters were as follows: postcontrast T1-weighted [repetition time (TR)/echo time (TE)/TI 2300/2.98/900 milliseconds; flip angle 9°; field of view (FOV) 256 × 240 mm; 176-208 slices; no slice gap; voxel size 1.0 × 1.0 × 1.0 mm] after intravenous injection of 9 ml gadobutrol (Gadovist,1.0 mmol/ml; Bayer, Leverkusen, Germany); T2-weighted (TR/TE 4840-5470/114 milliseconds; refocusing pulse flip angle 150°; FOV 220 × 165 mm; 23-26 slices; 0.5-mm slice gap; voxel size of 0.7 × 0.7 × 5.0 mm); DSC (TR/TE 1500/30 milliseconds; flip angle 90°; FOV 192 × 192 mm; 19 slices; slice gap 1.5 mm; voxel size of 2.0 × 2.0 × 5.0 mm) with 9 ml gadobutrol (Gadovist 1.0 mmol/ml) followed by a 20-ml saline flush administered via a power injector at 5 ml/s. DTI was acquired before contrast imaging using a single-shot echo-planar sequence (TR/TE 8300/98 milliseconds; flip angle 90°; FOV 192 × 192 mm; 63 slices; no slice gap; voxel size 2.0 × 2.0 × 2.0 mm; 12 directions; *b* values: 350, 650, 1000, 1300, and 1600 s/mm^2^; imaging time: 9 minutes 26 seconds). CSI utilized a semi-LASER sequence (TR/TE 2000/30-035 milliseconds; flip angle 90°; FOV 160 × 160 mm; voxel size 10 × 10 × 15-20 mm). PRESS excitation was selected to encompass a grid of 8 rows × 8 columns on T2-weighted images.

### Treatment and Evaluation of Response

Tumor resection was performed with the guidance of neuronavigation (StealthStation, Medtronic, Fridley, MN) and 5-aminolevulinic acid fluorescence (5-ALA, Medac, Stirling, UK) for maximal safe resection. Chemoradiotherapy regimen was determined after surgery by the multidisciplinary team according to patient postoperative status. Extent of resection was assessed according to the postoperative MRI scans within 72 hours as complete resection, partial resection of enhancing tumor, or biopsy [11]. All patients were followed up according to the criteria of Response Assessment in Neuro-Oncology [12], incorporating clinical and radiological parameters. Patient survival was analyzed for overall survival (OS) and progression-free survival (PFS). The latter was made retrospectively in some patients to avoid the issue of pseudoprogression, where new contrast enhancement appeared within the first 12 weeks after completing chemoradiotherapy.

### Image Processing

DSC data were processed and rCBV maps were generated after leakage correction using NordicICE (Nordic Neuro Lab, Bergen, Norway), during which an arterial input function was automatically defined. For each subject, all MR images were co-registered to T2-weighted images with an affine transformation using the linear image registration tool (FLIRT) functions in FSL [13].

The superimposed ^1^H MR spectroscopy data were analyzed using LC Model as described previously [14]. Briefly, only CSI voxels within tumor regions were included for analysis. All spectra were assessed for artifacts [15]. The quality and reliability of the ^1^H spectra were evaluated using Cramer-Rao lower bounds, with values greater than 20% discarded. A spectroscopic measure of lactate (Lac) was calculated as a ratio to the total creatine (Cr) [16]. To account for the different spatial resolution of T2 and CSI imaging, T2 pixels were projected to CSI space according to the spatial coordinates in MATLAB 2017b (The MathWorks, Inc., Natick, MA). Only CSI voxels completely in tumor region were included for further analysis.

### Regions of Interest

**Figure 1:**
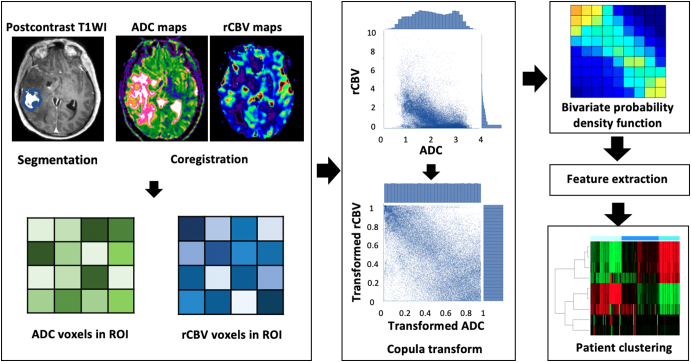
Study design. All images are co-registered before tumor regions are manually segmented from postcontrast T1-weighted images (T1WI). Voxels are then extracted from both ADC and rCBV maps. Empirical copula transform is performed on the joint distribution of ADC and rCBV voxels, which is then discretized before extracting second-order features from the matrix. These features are used in patient clustering to reveal patient subtypes.

The study design is illustrated in Figure 1. Tumor regions of interest (ROIs) were manually segmented using 3D slicer v4.6.2 (https://www.slicer.org/) by a neurosurgeon with >8 years of experience (C.L.) and a researcher with >4 years of brain tumor image analysis experience (N.R.B.) on the postcontrast T1 images. An interrater reliability testing was performed using Dice similarity coefficient scores to assure consistency among observers. For each individual patient, ROIs of normal-appearing white matter were manually segmented from the contralateral white matter and used as normal controls. The ADC and rCBV images were normalized by dividing by the mean value in the contralateral normal-appearing white matter.

### Copula Transform and Patient Clustering

We applied the copula transform to the ADC and rCBV maps on each patient individually, with no outliers removed. A discrete feature extraction was then applied. The extracted features included Energy, Contrast, Entropy, Homogeneity, Correlation, SumAverage, Variance, Dissimilarity, and AutoCorrelation [17]. A hierarchical clustering, using the complete method, was then performed on the patients based on the extracted features. To find the most stable and unambiguous patient clustering, we varied the number of clusters from 2 to 10. The optimal number of clusters was selected according to the majority vote among the 26 indices as implemented in the “Nbclust” package in R [18]. An R package, “XXXX,” for the implementation of the empirical copular transform and feature extraction was published online (https://github.com/XXX).

### Leave-One-Out Cross-Validation of the Clustering

A leave-one-out cross-validation (LOOCV) procedure was applied for constructing and validating the patient clusters. The clustering step was repeated by leaving one patient out of the cohort at each repetition. The consensus analysis was performed based on the clustering results from the LOOCV approach. A consensus matrix M was calculated, where M (*i, j*) indicates percentage of times that the patients *i* and *j* were clustered together across the dataset perturbations.

### Statistical Analysis

All analyses were performed in RStudio v3.2.3 (RStudio, Boston, MA). The clinical characteristics and CSI data of the clusters were compared with Kruskal-Wallis rank sum test using the Benjamin-Hochberg procedure to control the false discovery rate in multiple comparisons. Kaplan-Meier and Cox proportional-hazards regression analyses were performed to evaluate patient survival. Survival analysis was based on the subset of patients who received concurrent temozolomide (TMZ) chemoradiotherapy followed by adjuvant TMZ postoperatively. Cox proportional-hazards regression was performed, accounting for relevant covariates, including O-6-methylguanine-DNA methyltransferase (MGMT) methylation, isocitrate dehydrogenase-1(IDH-1) mutation, sex, age, extent of resection, and contrast-enhancing tumor volume. Patients who were alive at the last known follow-up were censored. The hypothesis of no effect was rejected at a two-sided level of .05.

## Results

### Patient Population

A total of 136 patients were recruited for preoperative MRI scan and surgery. After surgery, 115 (84.6%) glioblastoma patients (mean age 59.3 years, range 22-76 years, 87 males) were histologically confirmed. Of the 115 patients, 84 (73.0%) postoperatively received concurrent TMZ chemoradiotherapy followed by adjuvant TMZ (Stupp protocol). Other patients received short-course radiotherapy (17.4%, 20/115) or best supportive care (9.6%, 11/115) due to their poor postoperative performance status. Survival data were available for 80 of 84 (95.2%) patients as 4 (4.8%) patients were lost to follow-up.

Interrater reliability testing of ROIs showed excellent agreement between the two raters, with Dice scores (mean ± standard deviation [SD]) of 0.85 ± 0.10.

### Patient Clustering

**Figure 2:**
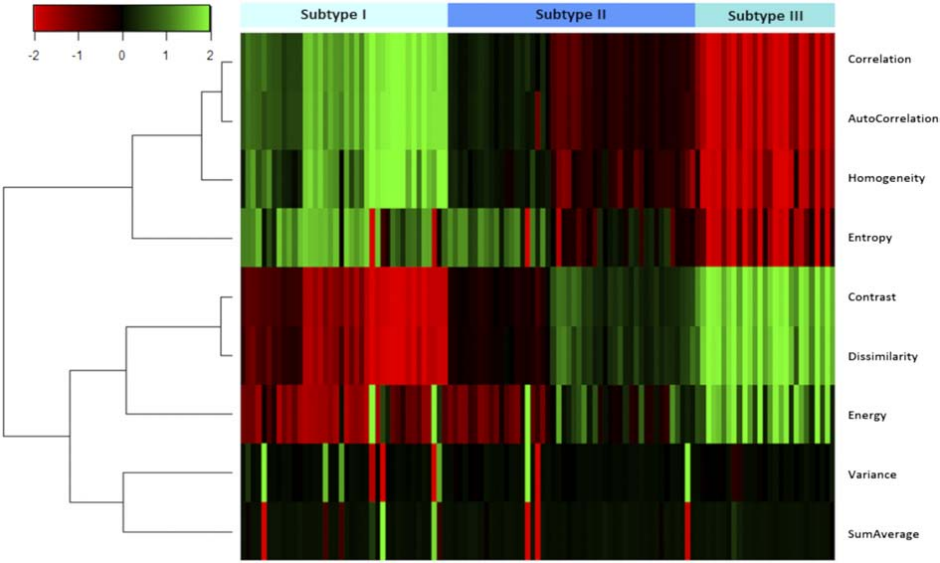
Patient clustering. Three patient clusters are identified using the features extracted from the joint distribution matrix of copula-transformed ADC and rCBV.

**Figure 3:**
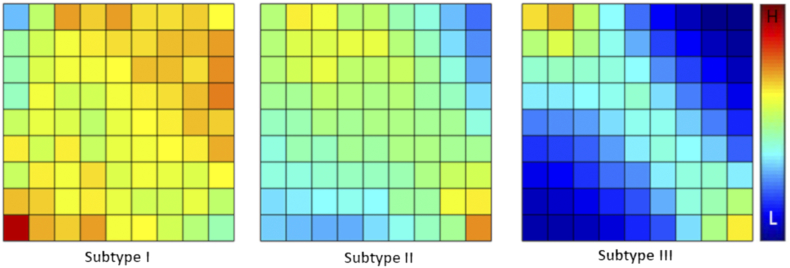
Average joint distribution matrices of three subtypes. The joint distribution of transformed ADC and rCBV values is discretized into a 10 × 10 joint distribution matrix for each patient. This figure shows the average matrix for each patient subgroup. Particularly, Subtype I displayed a most uniform joint distribution, and Subtype III displayed a most diagonalized joint distribution.

Based on the quantitative features characterizing the copula of ADC and rCBV, three patient clusters were identified through the hierarchical clustering, containing 40 patients (35%), 48 patients (42%), and 27 patients (23%) respectively (Figure 2). The average discretized matrices of ADC-rCBV joint distribution of three subtypes are demonstrated in Figure 3. Among the three subtypes, subtype I displayed a most uniform joint distribution, and subtype III displayed a most diagonalized joint distribution. Three subtypes showed no significant differences in clinical characteristics, as indicated in Table 1. However, the lactate levels of three subtypes were distinct (Table 2, Supplementary material 3). Particularly, Subtype III displayed a higher level of Lac/Cr ratio than Subtype II (*P* = .016) and Subtype I (*P* = .044).

**Table 1:** Clinical Characteristics of Subtypes

Variable	Subtype I (*n* = 40)	Subtype II (*n* = 48)	Subtype III (*n* = 27)	*P* Value
Age at diagnosis (range, years)	59 (33-76)	62 (38-75)	55 (22-73)	.261
Tumor volumes(cm^3^)	48.6 ± 31.4	41.0 ± 25.1	55.9 ± 33.1	.172
Male†	32	36	19	.663
Female†	8	12	8	
Complete resection†	30	30	17	.208
Partial resection†	7	16	9	
Biopsy†	3	2	1	
Methylated MGMT promoter*, †	20	17	11	.373
Unmethylated MGMT promoter*, †	19	30	14	
IDH-1 mutant†	1	3	3	.354
IDH-1 wild-type†	39	45	24	
Median OS (range)	403 (163-1077)	551 (78-1376)	407 (52-1333)	**.039**‡
Median PFS (range)	262 (93-758)	389 (25-1130)	244 (37-589)	**.025**‡

* MGMT promoter methylation status unavailable for four patients.

† Number of patients.

‡ Log-rank test.

**Table 2:** Lac/Cr Ratio of Subtypes

Subtype	Descriptive		Subtype II	Subtype III
	Mean ± SD	95% CI	*P* Value	*P* Value
Subtype I	12.9 ± 2.7	7.2 ± 18.6	.341	**.030**
Subtype II	9.8 ± 5.8	5.8 ± 13.8	/	**.006**
Subtype III	21.4 ± 3.4	14.3 ± 28.5	/	/

### LOOCV of Patient Subtypes

After the LOOCV, the co-occurrence consensus clustering matrix was computed. The results showed that three patient clusters generated from the unsupervised clustering were highly stable (Supplementary material 4). The mean values of the co-occurrence consensus clustering matrix were 0.91 for Subtype I, 0.95 for Subtype II and 0.98 for Subtype III.

### Survivals of Patient Subtypes

**Figure 4:**
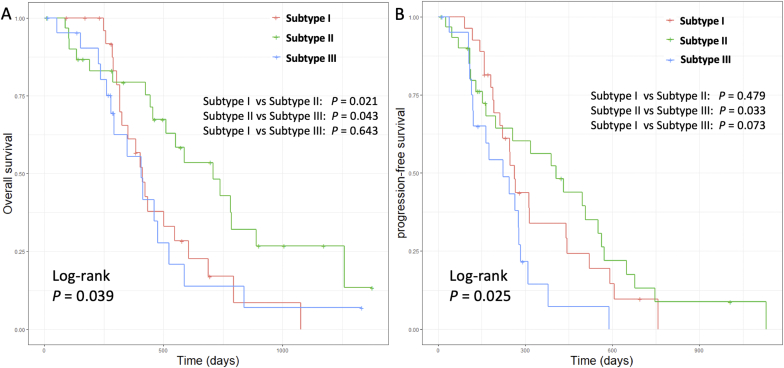
Survivals of patient clusters. Log-rank test shows that Subtype II displays better OS (*P* = .039) (A) and PFS (*P* = .025) (B) than Subtype I and Subtype III.

**Figure 5:**
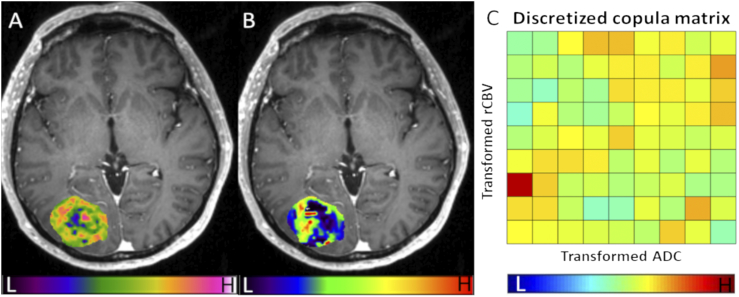
Case example of Subtype II. Pixel-wise ADC values (A) and rCBV values (B) are overlaid on postcontrast T1-weighted images. After the copula transform, the joint distribution is discretized (C). The matrix demonstrates a uniform distribution, which suggests a low interdependence of ADC and rCBV in this case.

Kaplan-Meier analysis using the log-rank test showed significantly different OS (*P* = .039) and PFS (*P* = .025) (Table 1, Figure 4) for the three identified subtypes. The Cox regression models (Table 3) accounted for all relevant clinical covariates. In the multivariate modeling of PFS, Subtype I showed significantly worse survival than Subtype II [hazard ratio (HR) = 1.992, *P* = .046]. Subtype III also showed significantly worse survival than Subtype II (HR = 3.062, *P* = .009). Extent of resection (HR = 2.710, *P* = .007) and MGMT methylation status (HR = 0.532, *P* = .025) significantly affected PFS. In the multivariate model of OS, Subtype I showed significantly worse survival than Subtype II (HR = 3.042, *P* = .003). The survival of Subtype III was not significantly different from Subtype II. Extent of resection (HR = 2.691, *P* = .011) and tumor volume (HR = 1.019, *P* = .001) significantly affected OS. Figure 5 demonstrates a case example of Subtype II.

**Table 3:** Survival Modeling

Factor	PFS						OS					
	Univariate			Multivariate			Univariate			Multivariate		
	HR	95% CI	*P* Value	HR	95% CI	*P* Value	HR	95% CI	*P* Value	HR	95% CI	*P* Value
Age	1.004	0.979-1.029	.758	1.027	0.994-1.062	.106	1.000	0.974-1.027	.988	1.004	0.971-1.038	.812
Sex (M)	1.555	0.923-2.618	.097	1.807	0.976-3.346	.060	1.243	0.695-2.222	.464	1.242	0.624-2.471	.537
Extent of resection	2.821	1.556-5.114	**.001**	2.710	1.321-5.560	**.007**	2.040	1.132-3.676	**.018**	2.691	1.259-5.754	**.011**
MGMT promoter methylation status*	0.619	0.369-1.039	.069	0.532	0.306-0.924	**.025**	0.573	0.320-1.027	.061	0.565	0.307-1.040	.067
IDH mutation status	0.986	0.356-2.733	.978	0.936	0.270-3.246	.917	1.038	0.369-2.926	.943	1.066	0.286-3.973	.925
Tumor volume†	1.005	0.996-1.015	.297	1.002	0.991-1.012	.742	1.018	1.008-1.029	**.001**	1.019	1.008-1.030	**.001**
Subtype I	1.267	0.701-2.289	.433	1.992	1.011-3.925	**.046**	2.017	1.051-3.873	**.035**	3.042	1.453-6.367	**.003**
Subtype III	2.389	1.240-4.602	**.009**	3.062	1.327-7.062	**.009**	2.089	1.092-4.386	**.027**	1.857	0.790-4.367	.156

* MGMT promoter methylation status unavailable for 2 patients.

† Contrast-enhancing tumor volume.

## Discussion

In this study, we characterized the interdependence of ADC and rCBV using the copula transform and evaluated the clinical significance of the interdependence in patient outcomes. The results showed that the interdependence of ADC and rCBV may provide information to evaluate the tumor microenvironment associated with patient prognosis.

Tumor microstructure estimated from diffusion imaging and vasculature estimated from perfusion imaging can describe key characteristics of solid tumor. Although evidence suggests that combining imaging modalities can identify tumor habitats responsible for treatment failure [8], a systematic method to investigate the interdependence of modalities is lacking. Previous studies have validated the robustness of the copula transform in estimating nonlinear correlation in multimodal neuroimaging data analysis [19]. Here we leveraged the copula transform to extract the joint distribution matrix of ADC and rCBV. The second-order statistics calculated from the joint distribution matrix can yield an array of measures for patient characterization. The resultant patient subtypes showed no significance in clinical factors but demonstrated significance in patient outcomes, suggesting that the interdependence of perfusion and diffusion imaging may offer information complementary to clinical factors.

The second-order features of ADC-rCBV joint distribution in Subtype III demonstrated the diagonalized pattern (Figure 3), suggesting the higher interdependence between vasculature and microstructure. Correspondingly, this subtype had a higher lactate than the other two subtypes, indicating a more hypoxic microenvironment. Interestingly, although Subtype I showed the most uniform joint distribution and therefore the least interdependent vasculature and microstructure, the survival of this subtype was worse than Subtype II. The higher lactate level of Subtype I implies a more hypoxic microenvironment than Subtype II. This finding suggests that both high interdependence and low interdependence between vasculature and microstructure are associated with more hypoxic tumor microenvironment and more invasive phenotypes, which may imply the nonlinear correlation between tumor cellularity and vascularity. A possible explanation could be that Subtype III may represent a highly proliferative phenotype with an unmet oxygen demand leading to global hypoxia, while Subtype I may have a less coupled microvasculature and microstructure leading to subregional hypoxia. The hypoxia in both subtypes could result in treatment resistance and poorer outcomes.

Our findings have clinical significance. The subtypes revealed by the interdependence between perfusion and diffusion may give insights that are potentially relevant for treatment strategy. Our findings showed that both high interdependence and low interdependence in tumor vasculature and microstructure were associated with hypoxia, which may cause resistance to adjuvant therapy. Cytoreductive surgery may be more crucial in these phenotypes. Future studies using a prospective cohort study design is needed to validate the clinical significance.

Previous studies have demonstrated the utility of classic radiomics analysis of single modality in patient stratification [20], [21]. Our proposed method could be further integrated with classic radiomics analysis in several regards. Firstly, the copula transform framework could be applied to a single modality as a normalization method, which could eliminate the acquisition uncertainty from different MRI sequences and scanners. Moreover, the features from the joint distribution of multiple modalities could be integrated with classical texture features from single modalities for tumor characterization. Further, our current study focused on the characterization of intertumoral heterogeneity. In our future study, this method could be integrated with habitat imaging to characterize the intratumoral heterogeneity [22], [23] by investigating the interdependency within habitats.

Our approach had limitations. Firstly, the resolution of CSI was lower than the resolution of the anatomical imaging, and ^1^H MR spectroscopy voxels were, therefore, larger than rCBV and ADC voxels. Secondly, our findings have not been validated in another independent validation cohort. Thirdly, our findings need further biological validation. Radiogenomics has been shown to unravel tumor phenotypes, which could possibly validate our results. Lastly, to reduce complexity, i.e., the spatial information–related noise, we applied discretization to the copula-transformed joint matrix in this study. Our future work will focus on feature extraction technique that incorporates the weight and continuous information of copula matrix.

## Conclusion

The interdependence between perfusion and diffusion imaging offers useful information that could potentially be used for evaluating the tumor microenvironment and glioblastoma patient stratification. This method could be extended to include more imaging modalities in future studies, with the advantage of copula transform in multidimensional distributions.

The following are the supplementary data related to this article.

Supplementary material 1 Theory. Supplementary material 1

Supplementary material 2 Flowchart demonstrating patient inclusion. A total of 136 patients were prospectively recruited for preoperative scanning and then underwent surgery. Postoperative pathology confirmed 115 patients with glioblastoma diagnosis, and 21 patients were excluded. After surgery, 84 patients received concurrent and adjuvant temozolomide chemoradiotherapy (CCRT). Due to their poor postoperative performance, 20 patients received short-course radiotherapy (SCRT), and 11 patients received best supportive care (BSC). Eighty patients were included in survival analysis, and 4 patients were lost in follow up. Supplementary material 2

Supplementary material 3 Lac/Cr of three patient clusters. Lac/Cr ratio in Subtype III is significantly higher than Subtype I (*P* = .030) and Subtype II (*P* = .006). *Lac*, lactate; *Cr*, creatine. *: *P* <0.05; **: *P* < 0.01. Supplementary material 3

Supplementary material 4 LOOCV of patient clusters. Consensus analysis was performed based on the 115 clustering results obtained from the LOOCV. The mean value of the co-occurrence consensus clustering matrix is 0.91 for Subtype I, 0.95 for Subtype II, and 0.98 for Subtype III. Supplementary material 4
