# Supplementary material for: Decoding the Interdependence of Multiparametric Magnetic Resonance Imaging to Reveal Patient Subgroups Correlated with Survivals
Source: Neoplasia. 2019 Mar 31;21(5):442–9. doi: 10.1016/j.neo.2019.03.005 (PMC6444075; doi:10.1016/j.neo.2019.03.005)
Supplement: Supplementary material 1 — Theory. [file mmc1.docx]

**Supplementary material 1. Theory**

**1. Challenges in analyzing the inter-dependence of random variables.** The existing literature involving analysis of inter-dependencies commonly originate from the probabilistic interpretation of Canonical Correlation Analysis [1]. To be precise, let $X=(x_{1},\ldots,x_{N_{X}})$and $Y=(y_{1},\ldots,y_{N_{Y}})$ be random variables with dimensions $N_{X}$ and$N_{Y}\mathbb{\in N}$, respectively (i.e.,$X\in\mathbb{R}^{N_{X}}$), then the following inter-dependence model can be introduced:

$$\begin{aligned} Z\sim Mult(\theta),\text{where}(X,Y)|Z\sim\mathcal{N}_{N_{X}+N_{Y}}(\mu_{z},\Phi), \end{aligned}$$

where $\Phi$ represents the covariance matrix of variables $X$ and $Y$.

The normal distribution assumption in the above model fitting, however, may be challenged by severe model mismatch if the variables are non-normally distributed. Later, it was proposed that normal distribution assumption on variables could be relaxed by applying exponential family transforms [2]. However, since most exponential transforms are not available in multi-dimensional settings, such method is still impractical for multidimensional data.

One way to mitigate above limitations is to apply copula framework. The empirical copula can be used to prepare our data for later discrete feature extraction and clustering. Therefore we named our approach inter-dependence clustering via copula transform and discrete feature extraction.

**2.** **Empirical copula.** In probability theory, a copula is a normalized multivariate probability distribution that provides a framework to quantify the inter-dependence structure between random variables. This framework is particularly useful when the given random variables follow non-normal distributions. Moreover, it can be applied to higher dimensional data, which is difficult when using standard joint distributions.

We first recall the definition of the copula transform and Sklar's theorem which provides theoretical validation for the application of the copula transform [3].

**Definition.** A function $C$: $[0,1]^{N}\to[0,1]$ is a $N$-dimensional copula if $C$ is a joint cumulative distribution function (CDF) of a $N$-dimensional random vector on the unit cube $[0,1]^{N}$ with uniform marginal.

**Sklar’s Theorem**. Let $F_{i}(x):=Pr[X_{i}\leq x]$ are continuous marginal CDFs. Then, every multivariate cumulative distribution function

$$\begin{aligned} H(x_{1},\ldots,x_{N})=Pr[X_{1}\leq x_{1},\ldots,X_{N}\leq x_{N}] \end{aligned}$$

of a random vector $(X_{1},\ldots,X_{N})$ can be expressed in terms of its marginal $F_{i}(x_{i})=Pr[X_{i}\leq x_{i}]$ and a copula $C$, such that

$$\begin{aligned} H(x_{1},\ldots,x_{N})=C(F_{1}(x_{1}),\ldots,F_{N}(x_{N})). \end{aligned}$$

The copula $C$ describes the dependence structure between the variables $X_{1},\ldots,X_{N}.$A wide range of marginal distributions, such as exponential, gamma, beta, and empirical, can be included in Sklar’s theorem. However, since a standard joint distribution is challenging to be obtained from noisy MRI data, we used the empirical copula to estimate the distribution function, namely*:*

$$\begin{aligned} C(u_{1},\ldots,u_{N}):=\frac{1}{N}\overset{N}{\underset{i=1}{\sum}}\chi_{(U_{1}^{i}\leq u_{1},\ldots,U_{N}^{i}\leq u_{N})}. \end{aligned}$$

**3. Clustering model specification.** Copula examines the inter-dependence between random variables across the entire distribution. In particular, they capture the effects of higher-order moments, i.e., skewness or kurtosis. Although such effects might benefit the research in Geostatistical interpolation and spatial statistics, they may possibly cause issues of fat-tails and skewness of data distribution, and such effeteness may hence present unnecessary noises and cause mismatch in clustering, including:

1. The weight of the data value at each point might be altered. That is, some data points might be given undesired priorities by the copula transform.
2. Spatial information of the data might be changed. The copula transform is not an isometric transform [4] and hence the data matrix may get deformed after the copula is applied.

**4. Methodology design.** Here we propose to use discrete feature extraction to reduce the above-mentioned noises elements as the spatial information is reduced in discretization process [5]. Our cluster analysis consists of the following steps:

**Step 1.** By applying the *empirical copula* transform, we normalized the voxel values of ADC and rCBV to $[0,1]$ and extracted the inter-dependence structure of the transformed ADC and rCBV values.

**Step 2.** We discretized the resulting copula into a $10\times10$ matrix, in which each element $p\left( i,j \right)$ represented the *relative frequency* of voxels within ROI falling into the corresponding discretized value range:

$$p\left( i,j \right)= \frac{P(i,j)}{\sum_{i=1}^{10} i\sum_{j=1}^{10} p\left( i,j \right)} (i,j=1,2,\ldots,10)$$

where $P(i,j)$ is the number of voxels whose normalized ADC and rCBV value falls into

$\left[ \frac{i-1}{10},\frac{i}{10} \right]$ and $\left[ \frac{j-1}{10},\frac{j}{10} \right]$,

respectively.

The following quantities were also defined:

$$\mu_{i}=\sum_{i=1}^{10} i\sum_{j=1}^{10} p\left( i,j \right) , \mu_{j}=\sum_{j=1}^{10} j\sum_{i=1}^{10} p\left( i,j \right)$$

$$\sigma_{i}=\sum_{i=1}^{10} {(i-\mu_{i})}^{2}\sum_{j=1}^{10} p(i,j) , \sigma_{j}=\sum_{j=1}^{10} {(j-\mu_{j})}^{2}\sum_{i=1}^{10} p(i,j)$$

The characteristic metrics of the copula matrix were calculated as second-order features [6], which included Energy, Contrast, Entropy, Homogeneity, Correlation, SumAverage, Variance, Dissimilarity, and AutoCorrelation:

$$Energy=\sum_{i=1}^{10} \sum_{j=1}^{10} {p\left( i,j \right)}^{2}$$

$$Contrast=\sum_{i=1}^{10} \sum_{j=1}^{10} {(i-j)}^{2}p\left( i,j \right)$$

$$Correlation=\sum_{i=1}^{10} \sum_{j=1}^{10} \frac{\left( i-\mu_{i} \right)\left( j-\mu_{j} \right)p\left( i,j \right)}{\sigma_{i}\sigma_{j}}$$

$$Homogeneity=\sum_{i=1}^{10} \sum_{j=1}^{10} \frac{p\left( i,j \right)}{1+\left| i-j \right|}$$

$$Variance=\frac{1}{10 \times10}\sum_{i=1}^{10} \sum_{j=1}^{10} \left[ \left( i-\mu_{i} \right)^{2}p\left( i,j \right)+\left( j-\mu_{j} \right)^{2}p\left( i,j \right) \right]$$

$$SumAverage=\frac{1}{10 \times10}\sum_{i=1}^{10} \sum_{j=1}^{10} \left[ i p\left( i,j \right)+j p\left( i,j \right) \right]$$

$$Entropy=-\sum_{i=1}^{10} \sum_{j=1}^{10} p\left( i,j \right) {log}_{2}(p(i,j))$$

$$Dissimilarity=\sum_{i=1}^{10} \sum_{j=1}^{10} \left| i-j \right| p\left( i,j \right)$$

$$Autocorrelation=\sum_{i=1}^{10} \sum_{j=1}^{10} ij p\left( i,j \right)$$

**Step 3.** A hierarchical clustering of patients was then performed based on the above features.

**5. Remark.** We should point out that in **Step 2**, when performing discretization, we only counted the relative frequency. Firstly, as frequency counting is invariant regarding weight changes of the data value, the extracted matrix is not affected by the possible priority shifting of the copula transform. Secondly, since we only computed the relative frequency of required data points relative to the given regions of interest, the extracted matrix is again invariant regarding possible deformation of the data distribution through the copula transform.

**6. Performance evaluation.** To evaluate the sensitivity and robustness of our proposed method. We investigated whether this method is sensitive to both data noise and tumor ROI uncertainty in following experiments:

1) We added independent Gaussian noise with different standard deviation (?? = 0, 0.1 and 0.5) to the ADC and rCBV images;

2) We eroded and dilated tumor ROI with a margin distance of 2 mm respectively.

After both steps, we evaluated the value changes of discrete PDF matrix after copula transform. For comparison, we also implemented feature scaling normalization method (X_new_ = (X-X_min_)/(X_max_-X_min_)) . The results are demonstrated in Figure A1 & A2.


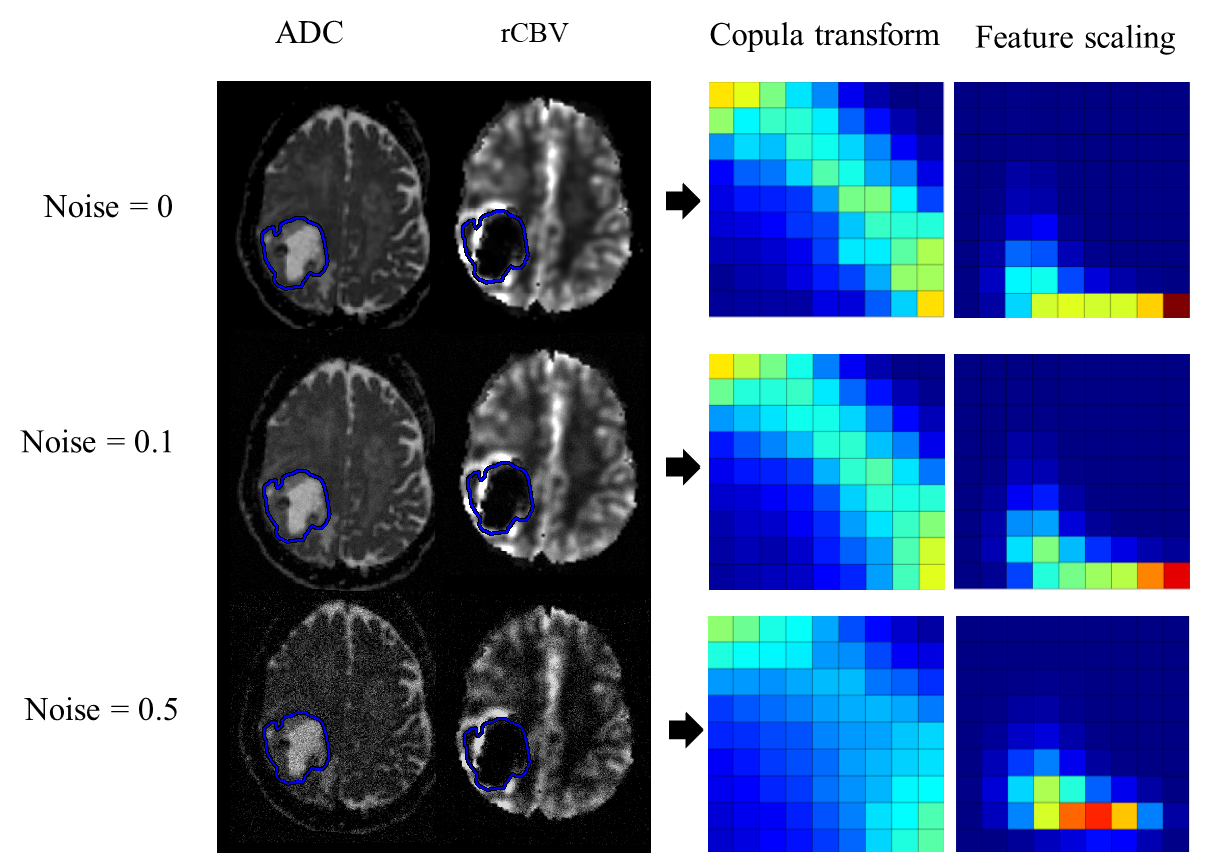


**Figure A1. Influence of Gaussian noise on transformation results**

As shown in Figure A1, due to the skewed distribution of rCBV, a significantly concentrated joint distribution of ADC and rCBV (characterized by low rCBV) can be observed after feature scaling normalization, which, therefore, may pose challenge to the characterization of the inter-dependence structure of ADC and rCBV. In contrast, the joint distribution after empirical copula transform is less effected by the concentrated low rCBV values and the data noise.


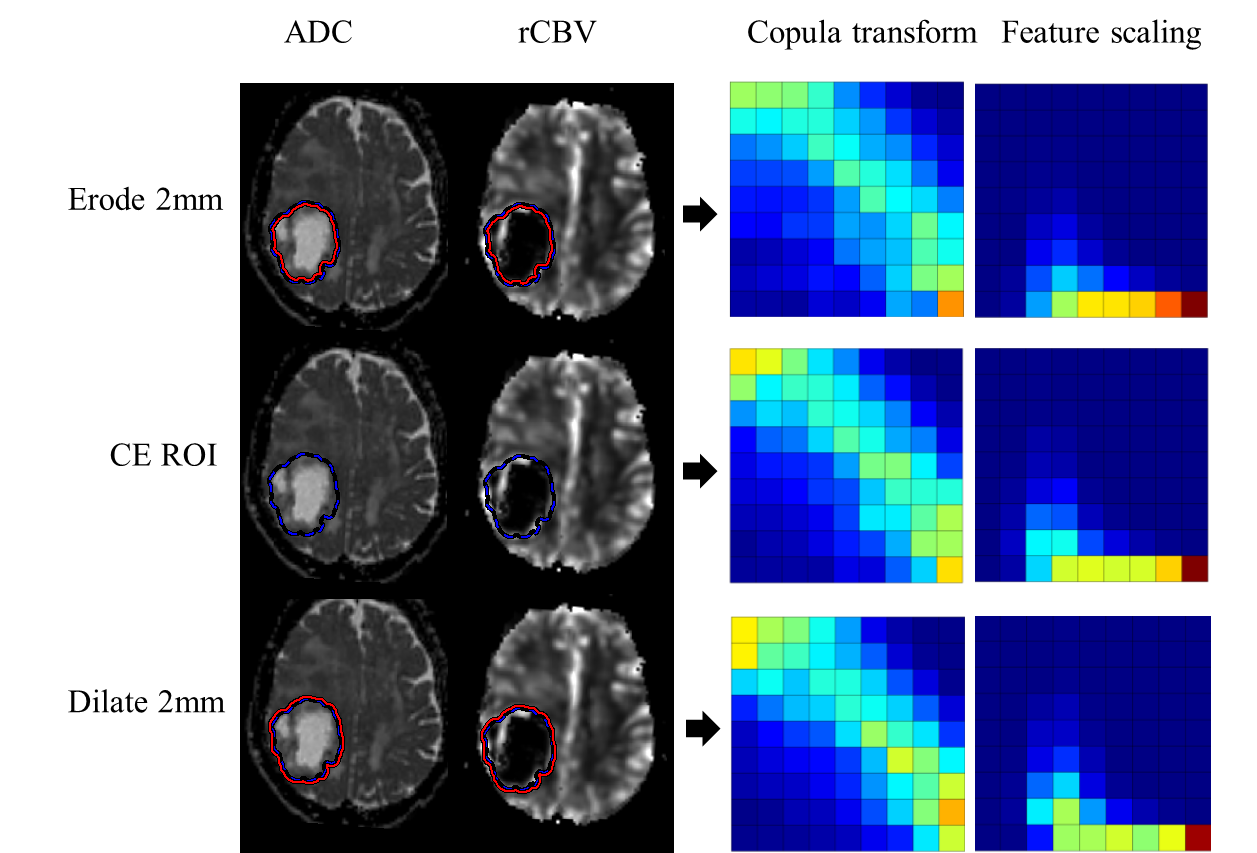


**Figure A2. Influence of differing ROI on transformation results**

As shown in Figure A2, the in-plane resolution of T2 space where manual segmentation was performed is 0.7mm × 0.7mm. We applied a 2mm erosion and 2mm dilation morphological operation respectively to simulate the tumor segmentation error. The results showed that the discrete matrix obtained from copula transform was not sensitive to the ROI contours (<10%).

After Gaussian noise were added and ROI were adjusted, the values in resulting discrete PDF matrix changed accordingly (Table A1). The results of our experiments showed that the copula transform yielded a more robust discrete matrix than feature scaling normalization, as indicated by the less value change with image noise applied.

| **Noise** | **Value change (Mean ± SD)** | |
| --- | --- | --- |
|  | Copula transform | Feature scaling |
| Gaussian noise = 0.1 | 0.001±0.001 | 0.002±0.005 |
| Gaussian noise = 0.5 | 0.003±0.003 | 0.013±0.029 |
| Dilated 2mm ROI | 0.002±0.002 | 0.002±0.005 |
| Eroded 2mm ROI | 0.001±0.001 | 0.002±0.005 |

**Table A1. Robustness of discrete joint distribution matrix under different noise and ROI**

In summary, our experiments demonstrated that the proposed approach is robust to the data noise and differing ROI.

[1] Bach FR, Jordan MI (2005). A probabilistic interpretation of canonical correlation analysis.

[2] Klami A, Virtanen S, Kaski S (2012). Bayesian exponential family projections for coupled data sources *arXiv preprint arXiv:12033489*.

[3] Sklar A (1973). Random variables, joint distribution functions, and copulas *Kybernetika* **9**, (449)-460.

[4] Lindenstrauss J, Tzafriri L (2013). *Classical Banach spaces II: function spaces*, Vol. 97. Springer Science & Business Media.

[5] Wiatowski T, Tschannen M, Stanic A, Grohs P, Bölcskei H (2016). *Discrete deep feature extraction: A theory and new architectures*. Editor (ed)^(eds): City, pp. 2149-2158.

[6] Haralick RM, Shanmugam K, Dinstein I (1973). Textural Features for Image Classification *Ieee T Syst Man Cyb* **Smc3**, 610-621.
