# Supplementary material for: Decoding the Interdependence of Multiparametric Magnetic Resonance Imaging to Reveal Patient Subgroups Correlated with Survivals
Source: Neoplasia. 2019 Mar 31;21(5):442–9. doi: 10.1016/j.neo.2019.03.005 (PMC6444075; doi:10.1016/j.neo.2019.03.005)
Supplement: Supplementary material 2 — Flowchart demonstrating patient inclusion. A total of 136 patients were prospectively recruited for preoperative scanning and then underwent surgery. Postoperative pathology confirmed 115 patients with glioblastoma diagnosis, and 21 patients were excluded. After surgery, 84 patients received concurrent and adjuvant temozolomide chemoradiotherapy (CCRT). Due to their poor postoperative performance, 20 patients received short-course radiotherapy (SCRT), and 11 patients received best supportive care (BSC). Eighty patients were included in survival analysis, and 4 patients were lost in follow up. [file mmc2.docx]

**
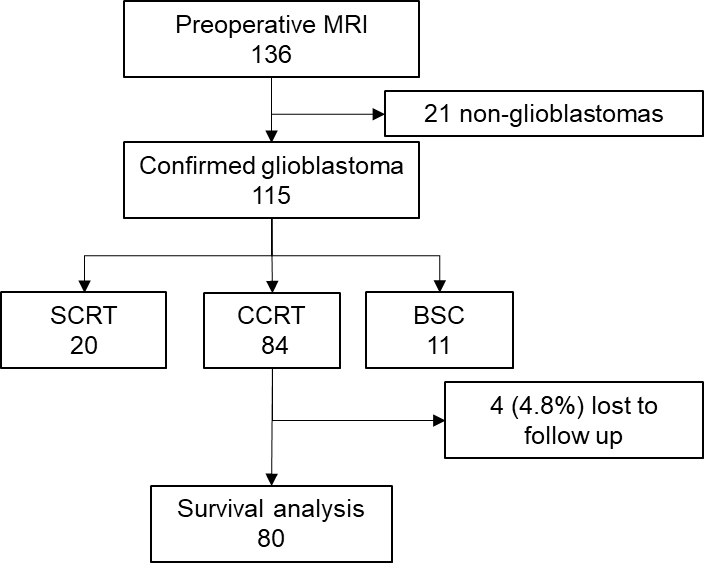
**

**Supplementary material 2. Flowchart demonstrating patient inclusion.** A total of 136 patients were prospectively recruited for pre-operative scanning and then underwent surgery. Post-operative pathology confirmed 115 patients with glioblastoma diagnosis and 21 patients were excluded. After surgery, 84 patients received concurrent and adjuvant temozolomide chemoradiotherapy (CCRT). Due to their poor post-operative performance, twenty patients received short-course radiotherapy (SCRT) and eleven patients received best supportive care (BSC). Eighty patients were included in survival analysis and four patients were lost in follow up.
