# Supplementary material for: Decoding the Interdependence of Multiparametric Magnetic Resonance Imaging to Reveal Patient Subgroups Correlated with Survivals
Source: Neoplasia. 2019 Mar 31;21(5):442–9. doi: 10.1016/j.neo.2019.03.005 (PMC6444075; doi:10.1016/j.neo.2019.03.005)
Supplement: Supplementary material 3 — Lac/Cr of three patient clusters. Lac/Cr ratio in Subtype III is significantly higher than Subtype I (P = .030) and Subtype II (P = .006). Lac, lactate; Cr, creatine. *: P <0.05; **: P < 0.01. [file mmc3.docx]

**
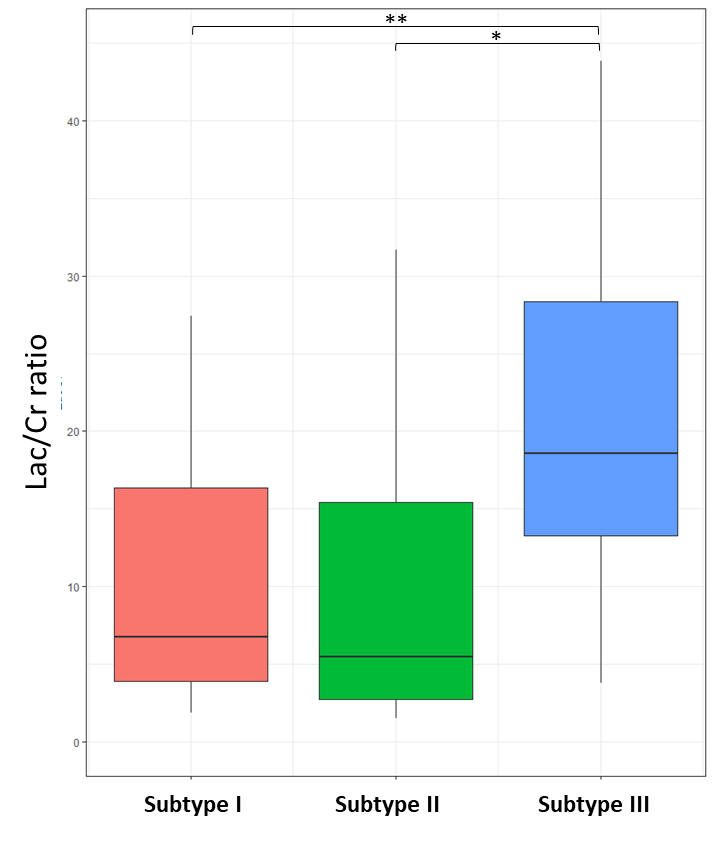
**

**Supplementary material 3. Lac/Cr of three patient clusters.** Lac/Cr ratio in Subtype Ⅲ is significantly higher than Subtype I (*P* = 0.030) and Subtype Ⅱ (*P* = 0.006). Lac: lactate; Cr: creatine; *: *P* <0.05; **: *P* < 0.01.
