# Supplementary material for: Decoding the Interdependence of Multiparametric Magnetic Resonance Imaging to Reveal Patient Subgroups Correlated with Survivals
Source: Neoplasia. 2019 Mar 31;21(5):442–9. doi: 10.1016/j.neo.2019.03.005 (PMC6444075; doi:10.1016/j.neo.2019.03.005)
Supplement: Supplementary material 4 — LOOCV of patient clusters. Consensus analysis was performed based on the 115 clustering results obtained from the LOOCV. The mean value of the co-occurrence consensus clustering matrix is 0.91 for Subtype I, 0.95 for Subtype II, and 0.98 for Subtype III. [file mmc4.docx]

**
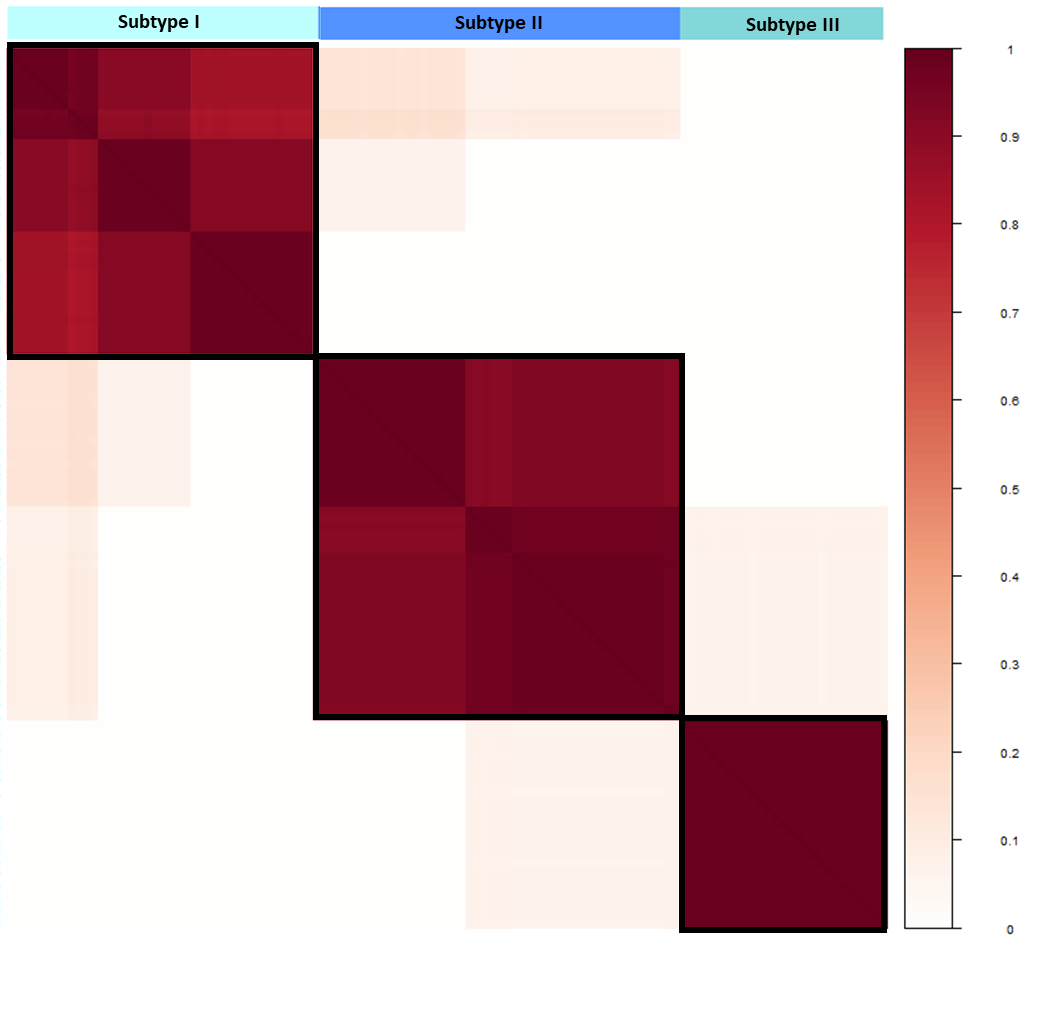
**

**Supplementary material 4. Leave-one-out cross validation of patient clusters.** Consensus analysis was performed based on the 115 clustering results obtained from the leave-one-out cross validation. The mean value of the co-occurrence consensus clustering matrix is 0.91 for Subtype I, 0.95 for Subtype Ⅱ and 0.98 for Subtype Ⅲ.
